# Genome-Wide Identification of Histone Modifications Involved in Placental Development in Pigs

**DOI:** 10.3389/fgene.2019.00277

**Published:** 2019-03-29

**Authors:** Kun Han, Ruimin Ren, Jianhua Cao, Shuhong Zhao, Mei Yu

**Affiliations:** Key Lab of Agricultural Animal Genetics, Breeding and Reproduction of Ministry of Education, College of Animal Science and Technology, Huazhong Agricultural University, Wuhan, China

**Keywords:** pig, placental development, H3K4me3, H3K27ac, transcriptome

## Abstract

Development of placental folds is a critical event affecting placental function in pigs because it can increase surface area for improvement in capillary density as gestation advances. However, the molecular mechanisms of the event are not well defined. Histone modifications have important roles in gene regulation. To investigate their effects on regulation of genes controlling porcine placental development, RNA-seq and ChIP-seq of porcine placental tissues from gestational days 50 (establishment stage of placental folds) and 95 (expanding stage of placental folds) were carried out in this study. The differentially expressed genes were identified and of which the down- and up-regulated genes are related to endoplasmic reticulum (ER) stress and angiogenesis, respectively. In addition, we mapped the genome-wide profiles of histone H3 lysine 4 trimethylation (H3K4me3) and histone H3 lysine 27 acetylation (H3K27ac), which are associated with transcriptional activation. A number of differential modification regions between the 2 gestational stages were identified and majority of them are those with increased signals of H3K4me3 (14,576 out of 16,931). Furthermore, we observed that the increase of H3K4me3 is significantly correlated with the elevated expression levels of the neighboring genes, and notably, these genes were enriched in pathways related to blood vessel formation and microvascular permeability. Taken together, the findings suggest important roles of histone modifications on placental remolding in response to developmental changes.

## Introduction

The placenta serves as a critical role for supporting the intrauterine fetal development and growth. Recent studies revealed that large numbers of genetic defects that leading to prenatal death are attributed to placental defects (Perez-Garcia et al., 2018). In addition, inadequate placental development is recently thought to be the important cause of the low efficiency of animal cloning by the somatic cell nuclear transfer (SCNT) technique (Yang et al., 2007; Whitworth et al., 2010; Matoba and Zhang, 2018). Unlike human placenta, pigs have a diffuse, epitheliochorial type of placenta (Leiser et al., 1998). Instead of invasion into the uterine wall, the porcine uterine epithelium remains intact throughout gestation (Dantzer, 1985; Vallet et al., 2009). The porcine trophoblast epithelial layer attaches to endometrial epithelial layer to form the epithelial bilayer around days 15–20 of gestation (gestation length for pigs is around 114 days). Thereafter, the trophoblast-endometrial epithelial bilayer develops placental folds to increase the maternal-fetal exchange surface area (Biensen et al., 1998). Around gestational day 50 (mid-gestation), the placental folds are established and in a steady-state stage. Then, from mid- to late-gestation, the placental folds increase in depth or complexity to further expand the exchange surface area (Vallet et al., 2013; Hong et al., 2014). In response to folds development, the density of the capillary adjacent to the folded epithelial bilayer becomes greater as gestation advances (Vallet and Freking, 2007; Che et al., 2016). Therefore, placental folds development is a critical contributor to maintaining the function of porcine placentas. Multiple molecular pathways and factors, such as heparanase (HPSE), were suggested to be involved in regulating placental development (Miles et al., 2009; Hong et al., 2014). However, mechanisms controlling gene expression in porcine placentas remain to be fully understood.

Histone modifications are important epigenetic marks of chromatin that regulate gene expression, thus play important roles in response to developmental and environmental changes (Turner, 2009). Among those histone modifications that have been discovered, methylation and acetylation are the two well characterized modifications to histones (Liang et al., 2004), and H3 is the most modified histone. During development of the human placenta, histone-H3 modifications are involved in regulation of genes associated with syncytialization and decidualization (Tamura et al., 2014; Fogarty et al., 2015; Shankar et al., 2015). In mice, changes in enhancer-associated histone modifications were found to be associated with trophoblast invasion (Tuteja et al., 2016). In addition, a complex epigenetic pattern of H3 modifications was detected to be related to early development of bovine embryo (Herrmann et al., 2013). Thus, acetylation and methylation of histone H3 have a significant effects on placental development and trophoblast differentiation. However, little is known about the effects of the histone modifications on development of porcine placentas. Trimethylation of histone H3 lysine 4 (H3K4me3) and acetylation of histone H3 lysine 27 (H3K27ac) are associated with transcriptional activation. H3K4me3 generally marks at promoters of transcribed genes, whereas H3K27ac marks both proximal and distal regions of transcription start site (TSS) (Barski et al., 2007; Wang et al., 2008; Heintzman et al., 2009; Creyghton et al., 2010). In this study, chromatin immunoprecipitation sequencing (ChIP-Seq) was performed to investigate the genome-wide profiles of H3K4me3 and H3K27ac in porcine placentas at gestational 50 (establishment stage of placental folds) and 95 (expanding stage of placental folds). Then integrated analysis of ChIP-Seq and RNA-Seq data was applied to identify the placental development related genes which are regulated by H3K4me3 and H3K27ac.

## Materials and Methods

### Samples Collection

All animal procedures were preformed according to protocols approved by the Ethic Committee of Huazhong Agricultural University. Chinese Meishan gilts were from the pig farm of Huazhong Agricultural University and naturally mated with a Meishan boar at the onset of estrus (Day 0) and again 24 h later, respectively. After the gilts were slaughtered on day 50 and 95 of pregnancy (*n* = 3–4 gilts/day of gestation), the uterus was removed immediately and each fetal placenta (chorioallantoic tissue) was collected and stored at -80°C for RNA extraction. In addition, the rectangular sections of the uteroplacental (including myometrium, endometrium, and placenta) were fixed immediately in fresh 4% paraformaldehyde for 24 h followed by paraffin embedding.

### Immunofluorescence Assay

Immunofluorescence assay was carried out according to protocol reported previously (Hong et al., 2016). In brief, sections were deparaffinized with xylene and rehydrated in a gradient alcohol and submitted to heat-induced epitope retrieval in 0.01 M sodium citrate buffer (pH 6.0) in a microwave for 15 min (three times, 5 min each time). After blocked with 5% bovine serum albumin (BSA) in PBS for 30 min in 37°C, sections were incubated with H3K4me3 antibody (1:50, ab8580, Abcam, United Kingdom) or with H3K27ac antibody (1:30, ab4729, Abcam, United Kingdom) at 4°C overnight and then with fluorescence-labeled secondary antibodies (1:100, BA1032, Boster Corporation, China) at 37°C for 30 min. Nuclear counterstaining was performed using DAPI. Slides were scanned by 3D HISTECH Pannoramic Midi scanner (3D HISTECH Ltd., Budapest, Hungary) and images were taken by CaseViewer software (3D HISTECH Ltd., Budapest, Hungary).

### RNA Sequencing

Total RNA was extracted from frozen placental tissue using RNeasy Plus Mini Kit (Qiagen, Germany) according to the manufacturer’s instruction. After removal of genomic DNA from RNA Preparations, RNA integrity number (RIN) was measured on Agilent 2100 Bioanalyzer (Agilent Technologies, Santa Clara, CA, United States) and only samples with RIN >8 were used for library construction. Thus, ten libraries (6 and 4 placentas from days 50 and 95 of gestation, respectively.) were constructed for RNA sequencing using Illumina platform (Novogene, Beijing, China). Paired end reads of 2 × 150 base pairs from each sample were generated by Illumina HiSeq X Ten sequencers. After removing adaptors and raw reads with PHRED <20, trimmed reads were obtained and mapped to the *Sus scrofa* genome assembly 11.1 using Hiseq2 (Kim et al., 2015) (Ensembl database version 91). Information about the number and quality of reads in RNA-seq data was listed in Supplementary Table 1. Reads counts for each gene from each sample was calculated using HTSeq (Anders et al., 2015). Differentially expressed genes were identified using DESeq2 (Love et al., 2014). The read counts were normalized using rlog function in the DESeq2 R package. The rld value of 10 was used to separate the genes with high and low expression. Gene Ontology (GO) analysis for the differentially expressed genes was carried out using g:Profiler (Reimand et al., 2016).

### ChIP Sequencing

Five placental samples were used for ChIP assay (3 and 2 placentas from days 50 and 95 of gestation, respectively). Antibodies H3K4me3 (ab8580, Abcam, United Kingdom) and H3K27ac (ab4729, Abcam, United Kingdom) were used in this study because they were ChIP grade and have been used in pigs (Seneda et al., 2008; Zhou et al., 2014). ChIP assay was carried out according to a revised protocol (Nelson et al., 2006). First, tissues were cross-linked with 1% formaldehyde for 15 min at room temperature. The reactions were terminated by addition of glycine. Second, tissues were lysed with 0.1 and 1% FA buffer (50 mM HEPES, pH 7.5; 150 mM NaCl; 1 mM EDTA; 1% Triton X-100; 0.1% sodium deoxycholate; 0.1% SDS and proteinase inhibitors) to obtain chromatin. The chromatin was sonicated with Ultrasonic Processor (Sonics VCX130). Third, the solubilized chromatin was immunoprecipitated with anti-H3K4me3 and anti-H3K27ac antibody as described above, respectively. After incubation overnight, the beads were washed and the captured IP-complex was eluted. Finally, the immunoprecipitated DNA (IP) and un-immunoprecipitated control (Input) were purified for construction of libraries and sequenced on an Illumina HiSeq X Ten platform (Novogene, Beijing, China).

Pre-processing of ChIP-seq raw reads was similar as that of RNA-seq used in this study. The cleaned data were mapped to the *Sus scrofa* genome assembly 11.1 using BWA-MEM (Li and Durbin, 2009). Then the duplicate reads and low quality reads (MAPQ <30) were filtered out using SAMtools (Li et al., 2009). Information about the number and quality of reads in ChIP-seq data was listed in Supplementary Table 1. To identify the H3K4me3 and H3K27ac peaks, replicates of ChIP-seq data were pooled and peaks of enriched occupancy relative to a background input were called using MACS2 (Zhang et al., 2008). The location of each peak was determined by using swine.gtf file (*Sus scrofa* genome assembly 11.1). Basic genomic region manipulations, including intersections and windows, were performed using BedTools v2.27.0 (Quinlan and Hall, 2010). Functional annotation and enrichment analysis of genes that locate near the predicted regions of H3K4me3 and H3K27ac was analyzed using ChIPpeakAnno (Zhu et al., 2010) and ChIPseeker (Yu et al., 2015). Differential modification regions were investigated according to previous reports (Pham et al., 2012; Tamura et al., 2014) with following steps: first, the modified regions were determined by BedTools-v2.27.0. Then, ChIP-seq counts were calculated. Peaks that are within 1 kb region were merged and the number of reads for both IP and INPUT samples in those regions were normalized by calculating RPKM (Reads per kilobase per million mapped reads). Third, the signal of each region was obtained by subtracting the INPUT RPKM value from the IP RPKM value. Regions increased or decreased more than 2-fold between days 50 and 95 of gestation were defined as differential modification regions.

### Quantitative Real-Time PCR and ChIP-qPCR

The RNA samples used for validation of RNA-seq data were the same as used in RNA sequencing. Total RNA was reverse-transcribed using PrimeScript RT Reagent Kit with gDNA Eraser (Takara Biomedical Technology, Shiga, Japan). The gene-specific primers were listed in Supplementary Table 1. The *GAPDH* gene was used as a control. ChIP-qPCR was performed on ChIP-enriched DNA to validate the ChIP-seq data. The input DNA sample was used as the negative control. Primers for ChIP-qPCR were designed within the H3k4me3-increased region determined by ChIP-seq assay (Supplementary Table 1). Real-time PCR was performed in triplicate using SYBR Premix Ex Taq (Takara Biomedical Technology, Shiga, Japan). The comparison between 2 samples from the 2 gestational stages was made using student’s *t*-test. A *P*-values of <0.05 was considered statistically significant.

## Results

### Transcriptome Profile of the Porcine Placentas

**FIGURE 1:**
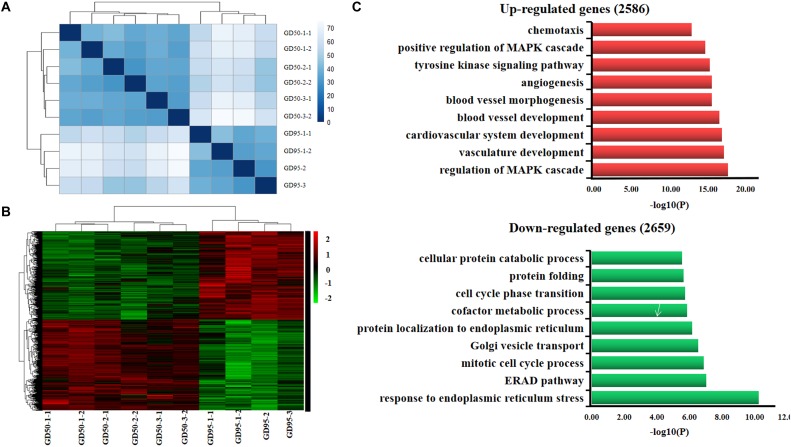
Gene expression profiling in porcine placentas on days 50 and 95 of gestation by RNA-seq. **(A)** Sample cluster analysis on differentially expressed genes (adjusted *P* value <0.01). **(B)** Hierarchical clustering heat map of differentially expressed genes (adjusted *P* value <0.01). **(C)** Gene ontology analysis of differentially expressed genes. GD50, day 50 of gestation; GD95, day 95 of gestation.

We investigated the transcriptome of porcine placentas (chorioallantoic tissues) on days 50 and 95 of gestation. Ten libraries (6 and 4 placentas from days 50 and 95 of gestation, respectively) were constructed and sequenced with Illumina paired-end sequencing technology. The cleaned reads were assembled into a total of 25,880 genes. Sample cluster analysis showed that the 10 samples were clearly separated according to the gestational days (Figure 1A). Subsequently, a total of 5,245 genes (including 2,586 up-regulated and 2,659 down-regulated genes, respectively) were found to be differentially expressed in placentas between days 50 and 95 of gestation (adjusted *P* value <0.01; Figure 1B and Supplementary Table 2). Gene ontology (GO) analysis showed that the most significantly overrepresented terms for the down-regulated genes are involved in endoplasmic reticulum (ER) stress (Figure 1C), transport and metabolism of nutrients whereas the most significantly overrepresented terms for the up-regulated genes are related to angiogenesis, cell-cell and cell-matrix interactions (Figure 1C).

### Expression Patterns of H3K4me3 and H3K27ac at Maternal-Fetal Interface in Pigs

**FIGURE 2:**
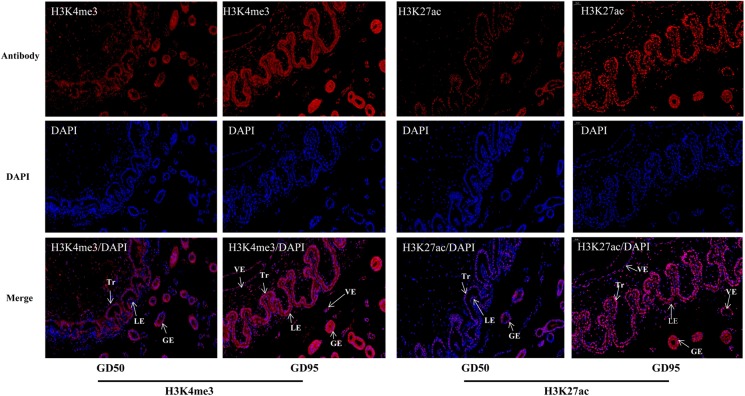
Immunofluorescence staining of H3K4me3 and H3K27ac antibodies in porcine placentas on days 50 and 95 of gestation. GD50, day 50 of gestation; GD95, day 95 of gestation; LE, endometrial luminal epithelium; GE, glandular epithelium; Tr, trophoblast; VE, vascular endothelium. Scale bars = 50 μm.

The immunofluorescence staining showed that nuclear staining for H3K4me3 and H3K27ac was observed in the luminal epithelium, glandular epithelium and vascular endothelial cell on the endometrial side, as well as the chorionic epithelium and vascular endothelial cell on the fetal side of the placenta. In addition, the staining intensity of H3K4me3 and H3K27ac was lower on gestational day 50 but increased on gestational day 95 (Figure 2).

### Genome-Wide Maps of H3K4me3 and H3K27ac in Porcine Placentas

**FIGURE 3:**
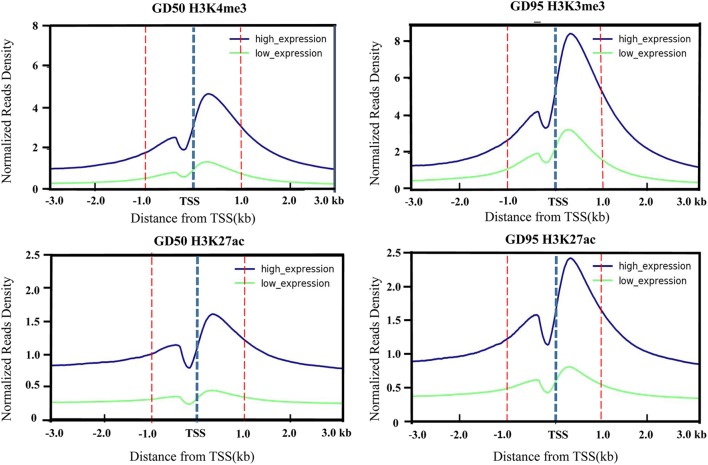
Profiles of the H3K4me3 and H3K27ac around TSS that were generated according to gene expression level. The H3K4me3 and H3K27ac signal densities were calculated as H3K4me3 and H3K27ac RPKM. Low-level and high-level expressed genes are represented by green and blue lines, respectively. GD50, day 50 of gestation; GD95, day 95 of gestation.

**FIGURE 4:**
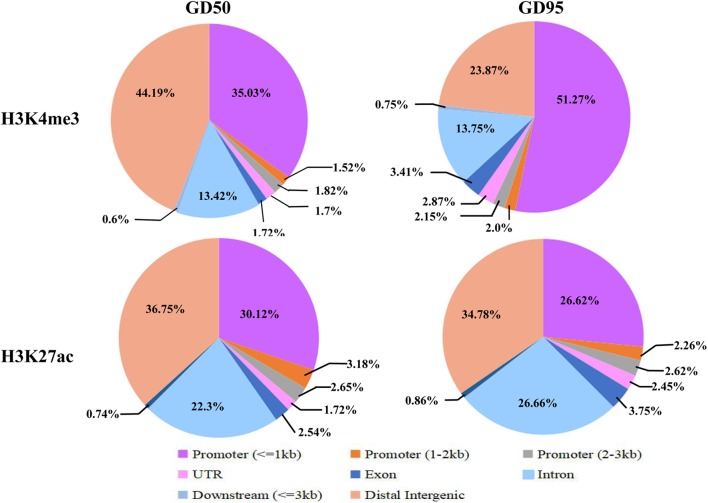
Distribution of H3K4me3 peaks in different genomic regions. Six genomic regions, including promoter (within ± 3 kb region from the TSS), intron, exon, downstream (3 kb downstream of the transcription termination site, TTS), UTR and distal intergenic, were classified according to the description in ChIPseeker (Yu et al., 2015). GD50, day 50 of gestation; GD95, day 95 of gestation.

To investigate the genome-wide landscape of histone modifications in porcine placentas on days 50 and 95 of gestation, we performed the ChIP-seq using antibodies against H3K4me3 and H3K27ac. The peak calling analysis was carried out by using model-based analysis for ChIP-seq with false discovery rate (FDR) <0.05. Totally, 39,671 H3K4me3 peaks, 24,281 H3K27ac peaks and 26,101 H3K4me3 peaks, 39,850 H3K27ac peaks were identified on days 50 and 95 of gestation, respectively (Supplementary Table 3). As shown in Figure 3, both H3K4me3 and H3K27ac signals were peaked within ± 1kb from the transcription start site (TSS). Subsequently, H3K4me3 and H3K27ac histone modification regions were classified into 6 genomic locations according to the description in ChIPseeker (Yu et al., 2015): promoter (within ±3 kb region from the TSS), exon, intron, downstream (3 kb downstream of the transcription termination site, TTS), UTR and distal intergenic. As shown in Figure 4, most of modification regions of H3K4me3 and H3K27ac were in proximal promoter region (≤1 kb) and distal intergenic regions. In addition, the distribution of H3K27ac marks in the 6 genomic regions was similar between the 2 gestational days, but a redistribution to TSSs (≤1 kb) was observed for H3K4me3 marks.

### Changes of H3K4me3 and H3K27ac Modifications in Porcine Placentas

**FIGURE 5:**
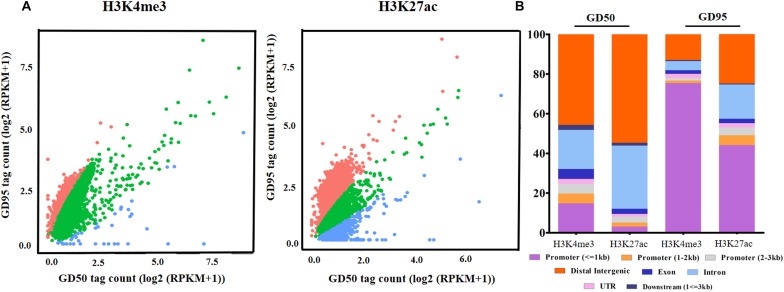
The altered H3K4me3 and H3K27ac modification regions in porcine placentas. **(A)** Changes of genome-wide H3K4me3 and H3K27ac histone modification statuses. The difference in signals between days 50 and 95 of gestation was evaluated by the scatter plot of log2 (RPKM +1). Red dots represent regions with increased signals of H3K4me3 and H3K27ac on day 95 of gestation. Blue dots represent regions with decreased signals of H3K4me3 and H3K27ac on day 95 of gestation. Green dots represent unchanged H3K4me3 and H3K27ac modification regions in placentas between days 50 and 95 of gestation. **(B)** The distribution of the differential histone modification regions in different genomic regions. GD50, day 50 of gestation; GD95, day 95 of gestation.

**FIGURE 6:**
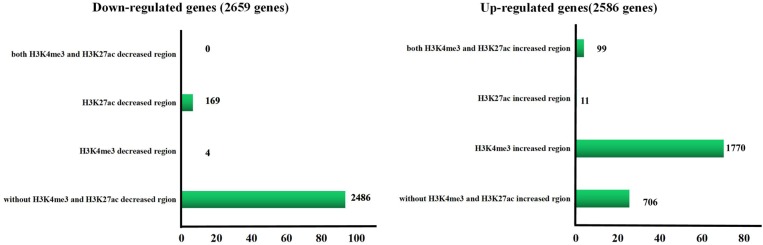
Number of the differentially expressed gene in different H3K4me3 and/or H3K27ac altered regions. GD50, day 50 of gestation; GD95, day 95 of gestation.

We defined the regions showing more than a 2-fold increase or decrease from gestational day 50 to 95 as the differential histone modification regions. Increased signals for H3K4me3 and H3K27ac were found in 14,576 and 921 regions, respectively, and decreased signals were detected in 130 and 1,304 regions, respectively (Figure 5A and Supplementary Table 4). As shown in Figure 5B, most H3K4me3 and H3K27ac marks with increased signals were in promoter regions, whereas most H3K4me3 and H3K27ac marks with decreased signals were located in intron and distal intergenic regions. Subsequently, the down-regulated/up-regulated genes were classified into 4 groups based on the statuses of histone modifications as described by Tamura et al. (2014): genes without H3K4me3- and H3K27ac- decreased/increased regions, genes with H3K4me3- decreased/increased regions, genes with H3K27ac- decreased/increased regions and genes with H3K4me3- and H3K27ac- decreased/increased regions. Of the 2659 genes that were down-regulated in placentas on gestational day 95, none of them have both H3K4me3- and H3K27ac- decreased regions, few genes had either the H3K4me3- or H3K27ac- decreased regions (H3K4me3: 4; H3K27ac: 169), thus most of them (2,486, 93.5%) had neither the H3K4me3- nor H3K27ac- decreased region (Figure 6 and Supplementary Table 5). However, of the 2586 genes with significantly up-regulated in placentas on gestational day 95, most genes (1,770, 68.4%) had the H3K4me3- increased regions and 99 genes had both the H3K4me3- and H3K27ac- increased regions (Figure 6 and Supplementary Table 5).

**FIGURE 7:**
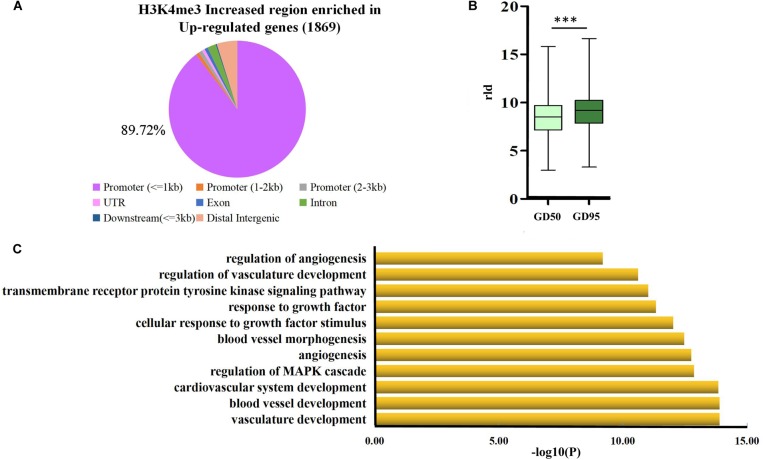
Analysis of the H3K4me3-increased region that were enriched in the proximal promoter region (≤1 kb) of up-regulated genes **(A)** genome-wide distribution of the increased signals of H3K4me3 that were found to be enriched in up-regulated genes (1,869 genes). **(B)** Box plot of rld expression value of genes that were up-regulated and affected by H3K4me3. ^∗∗∗^*P* < 0.001 **(C)** Gene ontology analysis of the up-regulated genes that were associated with the increase of H3K4me3 in the proximal promoter region (≤1 kb). GD50, day 50 of gestation; GD95, day 95 of gestation.

**FIGURE 8:**
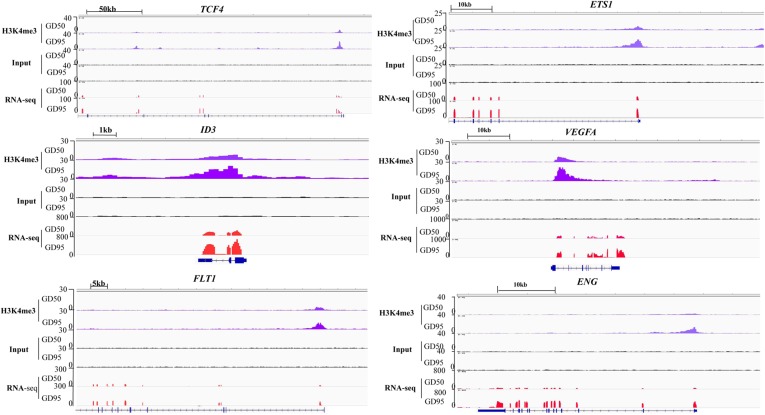
IGV (Integrative Genome Viewer) genome browser views of H3K4me3 modification patterns. GD50, day 50 of gestation; GD95, day 95 of gestation.

Therefore, we focused on investigation of the potential role of genes that had H3K4me3-increased regions during porcine placental development. Due to the observation that most of the H3K4me3-increased regions were enriched in the proximal promoter region (≤1 kb) (Figure 7A), expression levels of the up-regulated genes nearest to these proximal promoter region (≤1 kb) with H3K4me3 enrichment were quantified by using RNA-seq data. We found that the increase in mRNA levels of these up-regulated genes is significantly associated with the increase of H3K4me3 (Figure 7B). Thus, the findings suggest that the increase of H3K4me3 is involved in the up-regulation of gene expression in porcine placentas in response to developmental changes. Subsequently, GO term analysis was performed to investigate the function of the up-regulated genes nearest to these H3K4me3-increased regions (≤1 kb). The most significant GO terms were found to be associated with angiogenesis-related genes (Figure 7C), including *VEGFA* (vascular endothelial growth factor A), *FLT1* (Fms related tyrosine kinase 1, also known as vascular endothelial growth factor receptor-1, *VEGFR-1*), *ENG* (endoglin), *ID3* (inhibitor of differentiation-3), *ETS1* (ETS proto-oncogene 1) and *TCF4* (transcription factor 4) (Figure 8). The findings suggest that increase in the level of H3K4me3 at the proximal promoter region was mainly associated with the up-regulation of the nearby angiogenesis-related genes in porcine placentas during late gestation.

### Validation of the H3K4me3 Enriched Regions

**FIGURE 9:**
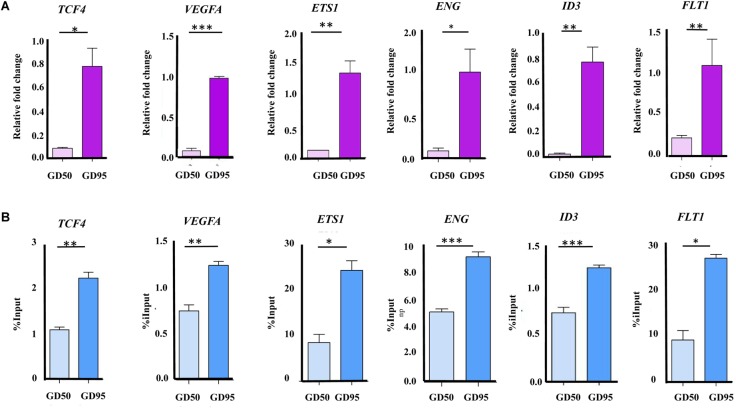
Validation of the RNA-seq and ChIP-seq data. **(A)** Validation of the RNA-seq data by using qRT-PCR. **(B)** Validation of the ChIP-seq data by using ChIP-qPCR. Data are represented as Mean+SEM, *n* = 3. ^∗^*P* < 0.05; ^∗∗^*P* < 0.01; ^∗∗∗^*P* < 0.001.

First, we confirmed that the expression levels of several angiogenesis-related genes (*VEGFA*, *FLT1*, *ENG*, *ID3*, *ETS1*, and *TCF4*) were increased significantly in porcine placentas from gestational days 50 to 95, which is consistent with the results from RNA-seq (Figure 9A). Second, we validated that enrichment in H3K4me3 in these angiogenesis-related genes was also increased significantly, which is in agreement with the ChIP-seq data (Figure 9B).

## Discussion

In this study, we identified genes that were differentially expressed in porcine fetal placentas during establishment and expanding stages of placental folds development. The RNA-seq data showed that the most enriched GO terms for up- and down-regulated genes are involved in angiogenesis and ER stress, respectively. Subsequently, we mapped the whole-genome profiles of H3K4me3 and H3K27ac in porcine fetal placentas. Finally, H3K4me3 was detected to have important effect on regulation of angiogenesis-related genes expressed in porcine placentas.

With the advancement of gestation, the porcine placenta undergoes remodeling to increase the placental efficiency through: (1) developing the placental folds to expand the maternal-fetal exchange surface area; (2) increasing the capillary density and permeability (Vallet et al., 2009). The RNA-seq data in the present study showed that most of up-regulated genes were those that are involved in blood vessel formation and microvascular permeability, such as *VEGFA*, *FLT1* and *ENG*. The finding is in agreement with previous reports revealed by using microarray technology (Zhou et al., 2009; Bischoff et al., 2013). On the other hand, we found that the most enriched GO terms for down-regulated genes are ER stress-responsive, such as response to ER stress (also called the unfolded protein response, UPR), ER-associated degradation (ERAD) pathway, and protein localization to ER (Figure 1C). The lumen of ER is the place where transmembrane and secreted proteins are made. In some conditions, such as nutrient deprivation, the demands of protein synthesis exceed the protein folding capacity of the ER, which will result in the accumulation of misfolded proteins in ER. The accumulation of improperly folded proteins active ER stress response to restore ER homeostasis by increasing the protein folding capacity of ER and degradation of improperly folded proteins (Schröder and Kaufman, 2005; Walter and Ron, 2011). Previously, we reported that secretion of carbohydrate macromolecules in placental trophoblast cells of Meishan pigs is much stronger on gestational day 50 but very few on gestational day 95, which implied an increased secreted protein synthesis on gestational day 50 as compared to gestational day 95 (Hong et al., 2017). From these findings, it can be speculated that the down-regulation of the ER stress-responsive genes indicates a restored ER homeostasis due to decreased secreted protein synthesis or/and enhanced placental surface area and vascularity.

The immunofluorescence stainings in this study revealed positive signals for H3K4me3 and H3K27ac in different components of porcine placenta. Then, we generated genome-wide ChIP-seq data for the 2 histone marks from the same placental samples used in RNA-seq. As expected, H3K4me3 and H3K27ac peaks were identified to be enriched around TSS and positively correlated with active gene expression. The findings are in agreement with the reported features of H3K4me3 and H3K27ac (Wang et al., 2008; Gifford et al., 2014). A number of differential histone modification regions for H3K4me3 or H3K27ac between the 2 gestational stages were identified (H3K4me3: 14,706; H3K27ac: 2,225). Of which, most of H3K4me3- and H3K27ac-decreased peaks were in distal intergenic regions, whereas H3K4me3- and H3K27ac-increased peaks were enriched in proximal promoter regions. H3K27ac is often found at promoter and distal intergenic regions (Gifford et al., 2014; Mas et al., 2018), while H3K4me3 generally marks at promoters near TSSs of expressed genes (Barski et al., 2007). The unexpected presence of distal intergenic H3K4me3 peaks could be explained by two possibilities that those peaks may either overlap with unannotated promoters or be associated with the activity of distal regulatory elements (Chen et al., 2015; Dhar et al., 2018; Medina-Rivera et al., 2018).

It is worth noting that the number of H3K4me3-increased regions (14,576) accounted for a large part of the altered H3K4me3 and H3K27ac signals (14,576 out of 16,931), indicating that H3K4me3 modification increased greatly throughout the genome in response to placental development changes. Consistent with the findings, majority of the down-regulated genes do not overlap with H3K4me3- and H3K27ac-deceased regions, but most of the up-regulated genes contain H3K4me3-increased regions that were located in proximal promoter regions (≤1 kb). This could be due to few of the down-regulated genes being H3K4me3 and H3K27ac targets, whereas H3K4me3 but H3K27ac marks target most of the up-regulated genes. In addition, our results showed that enrichment of H3K4me3 at proximal promoter regions was correlated with high expression of the neighboring genes. This is in agreement with the canonical feature that H3K4me3 primarily displays at promoters of active genes in somatic cells (Bannister and Kouzarides, 2011; Dahl et al., 2016; Yuen et al., 2017). Thus, compared to H3K27ac, H3K4me3 is more likely to be involved in the regulation of gene expression in porcine placentas.

GO analysis revealed that the up-regulated genes with higher levels of H3K4me3 in proximal regions were significantly enriched for several processes related to angiogenesis. Some blood vessel formation and microvascular permeability related genes, such as *VEGFA*, *FLT1*, *ENG*, *ID3*, *ETS1*, and *TCF4* were enriched in these pathways. *VEGFA* is well known for its roles in promoting angiogenesis, vascular permeability and cell migration (Shibuya and Claesson-Welsh, 2006). *FLT1* (also known as *VEGFR-1*) acts as a negative regulator by binding to *VEGFRA* and *VEGFRB* to establish a balance for vascular formation and permeability (Shibuya, 2006; Matsumoto and Ema, 2014). The broadly expressed transcription factor *ID3* was demonstrated as a mediator of *VEGFA* actions (Lyden et al., 2001; Sakurai et al., 2004). In addition, *ETS1* has been demonstrated to play an important role in regulating blood vessel formation by regulating expression of *FLT1*, *ID3*, and *ENG*, which is an endothelial cell marker (Li et al., 1999; Das et al., 2015; Wang et al., 2015). On the other hand, several reports indicated a role of *TCF4* in promoting neovascularization by interaction with β-catenin within nucleus (Jiang et al., 2015). All these genes were marked by H3K4me3 with a narrow peak in proximal promoter regions. Significant increases in H3K4me3 levels around proximal promoter regions of these genes were confirmed with ChIP-qPCR performed on porcine placentas from days 50 an 95 of gestation. Therefore, our results suggest that the occurrence of H3K4me3 peaks in the proximal promoter regions may contribute to regulating gene expression in porcine placentas.

## Conclusion

In conclusion, the present study obtained the whole-genome profiles of H3K4me3 and H3K27ac in porcine placentas collected from two key placental development stages. The increase of H3K4me3 were found to be associated with the up-regulation of genes related to angiogenesis. These findings suggest important roles of histone modifications in development of porcine placenta.

## Author Contributions

MY, SZ, and KH conceived and designed the experiments. KH and MY reviewed and discussed the results and wrote the manuscript. KH and RR performed the experiments. KH and JC analyzed the data.

## Conflict of Interest Statement

The authors declare that the research was conducted in the absence of any commercial or financial relationships that could be construed as a potential conflict of interest.
