# Supplementary material for: Genome-Wide Identification of Histone Modifications Involved in Placental Development in Pigs
Source: Front Genet. 2019 Mar 29;10:277. doi: 10.3389/fgene.2019.00277 (PMC6449610; doi:10.3389/fgene.2019.00277)
Supplement: Supplementary file 1 [file Table_1.doc]

Supplementary Table 1-1. RNA-Seq data quality

| Sample | Total reads | Mapping ratio (%) | Mapped reads |
| --- | --- | --- | --- |
| 170418-M50-L1 | 64740342 | 95.00% | 61501032 |
| 170418-M50-R1 | 61255666 | 95.04% | 58219223 |
| 170422-M50-L1 | 74865306 | 95.40% | 71423416 |
| 170422-M50-R2 | 69471338 | 95.24% | 66167734 |
| 170427-M50-L1 | 80505654 | 95.38% | 76790098 |
| 170427-M50-R1 | 81386592 | 95.32% | 77581272 |
| 170522-1-M95-L1 | 68174039 | 95.21% | 64911842 |
| 170522-1-M95-L3 | 76109667 | 95.41% | 72613334 |
| 170522-2-M95-R2 | 64737065 | 95.14% | 61588579 |
| 170625-M95-R1 | 70088764 | 95.06% | 66627602 |

Supplementary Table 1-2. ChIP-Seq data quality

| Sample | Total reads | Mapping ratio (%) | Duplicated reads ratio (%) | High quality reads (mapQ≧30) |
| --- | --- | --- | --- | --- |
| 170418-M50-H3K4me3 | 58482995 | 99.56% | 0.32 | 51994074 |
| 170418-M50-H3K27ac | 71352351 | 99.74% | 0.22 | 67738466 |
| 170418-M50-Input | 73134898 | 99.53% | 0.25 | 67650343 |
| 170422-M50-H3K4me3 | 66391446 | 99.26% | 0.00 | 57949660 |
| 170422-M50-H3K27ac | 63936405 | 99.60% | 0.23 | 59973020 |
| 170422-M50-Input | 58877635 | 99.70% | 0.26 | 58877634 |
| 170427-M50-H3K4me3 | 62658245 | 99.48% | 0.29 | 56656383 |
| 170427-M50-H3K27ac | 64414572 | 99.76% | 0.23 | 60553115 |
| 170427-M50-Input | 66673481 | 99.70% | 0.25 | 61546163 |
| 170522-1-M95-H3K4me3 | 67060747 | 100.00% | 0.22 | 63324067 |
| 170522-1-M95-H3K27ac | 67967165 | 100.00% | 0.28 | 61961286 |
| 170522-1-M95-Input | 75766077 | 99.24% | 0.00 | 69314372 |
| 170522-2-M95-H3K4me3 | 58912005 | 99.33% | 0.28 | 53899420 |
| 170522-2-M95-H3K27ac | 81847350 | 99.56% | 0.22 | 77530694 |
| 170522-2-M95-Input | 70873902 | 99.58% | 0.25 | 65733885 |
| 170625-M95-H3K4me3 | 59476817 | 99.55% | 0.25 | 56617863 |
| 170625-M95-H3K27ac | 71479323 | 99.68% | 0.22 | 67717154 |
| 170625-M95-Input | 61249495 | 99.53% | 0.25 | 55353056 |

Supplementary Table 1-3. List of oligonucleotide sequences used in this study.

qRT-PCR Primers

| Gene | Forward Primer (5'-3') | Reverse Primer (5'-3') |
| --- | --- | --- |
| *TCF4* | CCGCCAACAGACATTCACTC | TCCTGGGGTGGGTTCAAATC |
| *ID3* | CCAAACGACCTTCTGCCACT | GAGGCAGGGATTTGGGGAAG |
| *VEGFA* | ATCTTCAAGCCGTCCTGTGT | TCCTATGTGCTGGCCTTGGT |
| *FLT1* | GCAGAAGATTCGGGGGCTTA | CCGGGAAGGATGACACAGC |
| *ETS1* | TTCGTGGATTCTGCTGCGAG | TCATCCCAGAAGGGGTAGCA |
| *ENG* | TCTCATCTCGACTCTGGGGT | TGCGGTGATGAGCTTGACAG |

ChIP-qPCR Primers

|  | Forward Primer (5'-3') | Reverse Primer (5'-3') |
| --- | --- | --- |
| *TCF4* | AGCTGCCTGAGAACCGACT | CCGCAACCCTGTTTTGTAGG |
| *ID3* | ATCACAGTAACCACGGAGGC | TGCTTTCTGTGAGTTCGGGA |
| *VEGFA* | GTTCCCCGTTCCTCCAGTTT | CCGTCCATTCATCGATCCCA |
| *FLT1* | CCGATTCCTCAAACCGCATC | GCAGTCGAGGACCCACATAC |
| *ETS1* | GCGGCATAGCCCACTTGT | TGGAGAATCGGACGGACTTT |
| *ENG* | AGGGACGTTGGGGTTTTTCT | TCTGGGAACACACCGTTAGG |
